# Associations of perceived social support and positive psychological resources with fatigue symptom in patients with rheumatoid arthritis

**DOI:** 10.1371/journal.pone.0173293

**Published:** 2017-03-14

**Authors:** NeiLi Xu, Shuai Zhao, HongXia Xue, WenYi Fu, Li Liu, TianQi Zhang, Rui Huang, Ning Zhang

**Affiliations:** 1 The Second Clinical Academy of China Medical University, Shenyang, Liaoning, China; 2 The Department of Rheumatology at Shengjing Hospital of China Medical University, Shenyang, Liaoning, China; 3 Department of Social Medicine, School of Public Health, China Medical University, Shenyang, Liaoning, China; University of Texas Health Science Center at Houston, UNITED STATES

## Abstract

**Objective:**

This study aimed to assess the association between perceived social support (PSS) and fatigue and the roles of hope, optimism, general self-efficacy and resilience as mediators or moderators on PSS-fatigue association among Rheumatoid Arthritis (RA) patients in China.

**Methods:**

A multi-center, cross-sectional study was conducted withinpatients diagnosed with RA in northeast China, in which 305 eligible inpatients were enrolled. The Multidimensional Fatigue Inventory, Multidimensional Scale of Perceived Social Support, Herth Hope Index, Life Orientation Test Revised, General Self-Efficacy Scale and Ego-Resiliency Scale were completed. The associations of PSS, hope, optimism, general self-efficacy and resilience with fatigue and the moderating roles of these positive psychological constructs were tested by hierarchical linear regression. Asymptotic and resampling strategies were utilized to assess the mediating roles of hope, optimism, general self-efficacy and resilience.

**Results:**

The mean score of the MFI was 57.88 (SD = 9.50). PSS, hope, optimism and resilience were negatively associated with RA-related fatigue, whereas DAS28-CRP was positively associated. Only resilience positively moderated the PSS-fatigue association (B = 0.03, β = 0.13, P<0.01). Hope, optimism and resilience may act as partial mediators in the association between PSS and fatigue symptoms (hope: a*b = -0.16, BCa 95%CI: -0.27, -0.03; optimism: a*b = -0.20, BCa 95%CI: -0.30, -0.10; resilience: a*b = -0.12, BCa 95%CI: -0.21–0.04).

**Conclusions:**

Fatigue is a severe symptom among RA patients. Resilience may positively moderate the PSS-fatigue association. Hope, optimism and resilience may act as partial mediators in the association. PSS, hope, optimism and resilience may contribute as effective recourses to alleviate fatigue, upon which PSS probably has the greatest effect.

## Introduction

Rheumatoid arthritis (RA) is a common autoimmune disease, mainly manifested as chronic, symmetric and progressive multi-arthritis, with a prevalence of 0.3–1.0% worldwide [1] and nearly 0.37% in China [2]. Fatigue is regarded as an extra-articular symptom and is the most common complaint besides pain among RA patients, with prevalence of 42%-80% or even more [3]. One study found that 57% patients have identified fatigue as the most important problem [4]. In RA patients, fatigue is considered as a chronic, complex, unpredictable, frustrating and overwhelming experience, typically not associated with excessive exertion and poorly relieved by rest [3–6]. Its exact etiology remains barely understood but is considered to be multidimensional. In addition to the inflammation itself, other researches have concluded that social factors, gender, cognition and emotion, impaired sleep patterns, pain, and environmental factors may also be related to the onset of fatigue [7, 8]. Because fatigue is a symptom of subjective experience, self-assessment is the most commonly used descriptive tool. Fatigue is also often considered to be a multi-dimensional framework. For example, some consider fatigue consists of 5 dimensions: general fatigue, physical fatigue, reduced activity, reduced motivation and mental fatigue; meanwhile, some considers 4 dimensions: cognitive, physical, emotional and living with fatigue [9].With tremendous adverse consequences, fatigue penetrates every corner of daily routines and poses a substantial humanistic and financial burden on the overall quality of life, thus decreasing patients’ general well-being [1].

However, no specific pharmacological strategies have been discovered to produce meaningful improvements in the symptom to date. An 8-year follow-up concluded that the clinical influence of the severity of inflammation (for instance, inflammation markers and disease activity) on fatigue was actually quite small despite being statistically correlated [6]. Additionally, many studies have suggested that even when the inflammation was under control as indicated by low disease activities or achieving the desirable goal of remission, the fatigue wasn’t remarkably alleviated. Therefore, because no evidence exists to support the improvement by available medications, research on the psychosocial strategies may become a task of top priority to relieve RA-related fatigue [10, 11].Social support is defined as a helpful resource that can meet an individual’s urgent needs and is provided by a network of others, such as family members, friends, colleagues and other communities [12]. Social support may be divided into 2 groups: perceived social support (PSS), the subjective support that individuals may experience, emphasizing the individual's self-understanding, experience and feelings of social support from different sources; and enacted social support (ESS), which refers to the objective, practical and visible support, including direct physical assistance and assistance from a social network [13]. It has been suggested that PSS can play a greater role on physical health than ESS [14]. Social support directly and moderately exerts beneficial effects on the health-related quality of life in RA patients [8, 12]. One study found that approximately one-fifth of patients with RA had been on bad terms with their family and friends due to fatigue [15]. Another study concluded that RA patients usually considered themselves short of sufficient social support [16], which should highlight its impact on fatigue symptoms. According to a recent systematic review, several results have demonstrated that more social support indeed had a positive influence on fatigue outcomes in RA [17]. However, that conclusion haven’t been confirmed due to the small quantity of available relevant studies, indicating that further research is required.

As a consequence of emerging positive psychology in recent decades, beyond the limited positive effects of external social factors, some internal psychological constructs, such as hope, optimism, general self-efficacy and resilience, have been attributed increasing recognition of playing positive roles among clinical research and trials [18]. Further, social support can improve the characteristics of these positive psychological variables, to reduce the effects of these traits on social support.

Hope, as a positive cognitive-affective process, is considered to be multidimensional and dynamic; it refers to the ability of an individual to strive to realize their future goals despite a sense of uncertainty [19]. Many studies have demonstrated that an individual’s level of personal hope is related to mental development and health conditions, which could improve mental physical health. Hope is an important part of treatment process, by helping patients overcome difficulties and relieve stress, thereby alleviating the suffering. Perceived social support is one of the most important factors affecting the level of hope [20]. Hope may have a positive effect on outcomes of different disease populations [18, 21]. One study reported that those patients with greater feelings of hope suffered from less fatigue, demonstrating that bolstering hope may result in less fatigue in chronic illness [22].

Optimism refers to an individual’s tendency to maintain positive expectations of future events with an inclination towards believing that there is more good than bad in any situation [23]. Optimists tend to evaluate development trends and results of things with positivity [24]. Optimism is a positive subjective experience, and is an important subject in the field of positive psychology. According to Dr. Martin Seligman, optimism can be cultivated and is aimed at stimulating people's inner strength and good qualities to help people to maximize their potential and maintain an overall better condition. Developing an optimistic attitude has become one of the most important ways to improve person's health [25]. Researches have suggested that optimism might exert direct impacts on inflammatory and immune responses [26] and suggested possible associations between optimism and two rheumatic diseases: Ankylosing spondylitis and chronic low back pain, by potentially improving health-related quality of life, especially mental health [23].

According to Schwarzer, the definition for general self-efficacy is that an individual’s capacity to convince himself to achieve success by accomplishing one or a series of actions in various situations [27]. It refers to an individuals’ overall confidence when coming across challenges or confronting new things [28, 29]. People with higher general self-efficacy always set higher goals, make more effort and are reluctant to give up. It is considered to be an important psychological resource, and it directly and indirectly affects the physical and mental health-related of quality of life in patients with arthritis. Some researches have concluded that with lower self-efficacy, arthritis-related symptoms such as pain and fatigue become more severe [8, 17]. However, little similar research has been carried out in mainland China due to a lack of intervention programs for general self-efficacy [8].

Resilience is defined as a person’s capacity to adapt to anexisting situation when under pressure or confronted with a critical condition instead of stagnating in the original state [30], which has been a hot topic in positive psychology for many years. Psychologists believe that resilience is the main reason why patients with the same physical status have different feelings about their own quality of life and that better resilience can help patients to respond quickly to the physical and psychological adjustment in the face of disease [31]. Many researchers have suggested that resilience, as a dynamic process, is not always innate and may be learned in certain circumstances [32]. Moreover, acquired environment, such as education and intervention training, probably has a greater impact on individuals’ resilience than congenital genetics. For example, positive intervention can promote the improvement of the resilience and contribute to an individual's development [33]. Increasingly, researches have illuminated that resilience, contributecd as a defense mechanism or protective factor, is associated with emotional adjustment and could prevent patients from experiencing distress or at least reduce it [30, 34]. Additionally, resilience is related to fatigue in different disease populations [35, 36].Although the newly emerging field of positive psychology is under rapid development, almost none research has been conducted to evaluate the associations among PSS, positive psychological constructs and fatigue in patients with RA. Furthermore, if they are interrelated, little is known about the the specifics of how they interact with one another. More importance should be attached to PSS and psychological constructs as possibilities to alleviate symptoms of fatigue in RA.

Therefore, in accordance with the analysis above, the objective of the present study are as follows:

1. To assess the effect of PSS on fatigue among RA patients, simultaneously adjusting for related factors including demographic and clinical variables.
2. To assess whether hope, optimism, general self-efficacy and resilience moderate or mediate the association between PSS and fatigue.

## Materials and methods

### Study design and sample

From December 2014 to January 2016, a multi-center and cross-sectional study was conducted in successive inpatients diagnosed with RA. Patients were recruited at the Department of Rheumatology at Shengjing Hospital of China Medical University, Central Hospital of Benxi, General Hospital of Fushun Mining Bureau and Sujiatun Central Hospital of Shenyang, which are characterized as key medical centers that gathers substantial numbers of patients in Liaoning Province and even in northeast China. The protocol of research was inspected and authorized by the Committee on Human Experimentation of each hospital and complied with current ethical standards. Each patient signed the informed consent.

Patients diagnosed with RA based on ACR/EULAR 2010 met the inclusion criteria as follows: (1) ≥ 18 years of age; (2) had clear consciousness and cognition(be able to answer questions on persons, time and places within 30s); (3) were able to consciously complete questionnaires. The exclusion criteria included: (1) had a psychiatric history and intellectual injury(e.g. depression, anxiety, schizophrenia and other psychiatric disorders); (2) were taking psychotropic drugs; (3) were suffering from other severe illness(diseases which are at acute stage threatening patients’safety of life and have a serious impact on fatigue besides RA).

All eligible patients enrolled in the research voluntarily and anonymously. They indicated their understanding the objectives and procedures of the research as described by Rheumatologists-in-charge and relevant residents. Each patient signed the informed consent form. Questionnaires were completed by the patient himself or with the help of others under the guidance of investigators. Specifics of patients’ conditions were evaluated by physicians including duration of early morning stiffness (EMS), swollen joint counts (SJC) and tender joint counts (TJC). The clinical data was collected from medical records comprising C-reaction protein (CRP) and erythrocyte sedimentation rate (ESR).

There were 325 patients recruited as potential subjects at the initial stage, and 12 respondents subsequently refused to cooperate. Eight respondents with more than 30% of missing information were removed, with an effective response rate of 93.8%. Ultimately, 305 subjects qualified for the research.

### Measurement of fatigue

The degree of fatigue was measured by the Chinese version of the Multidimensional Fatigue Inventory (MFI-20) [37]. The MFI, containing 20 items with scores ranging from 20 to 100, comprised of 5 diverse dimensions: general fatigue, physical fatigue, reduced activity, reduced motivation and mental fatigue with 4 items each. The score for each item is in accordance with the degree of each individual’s specific condition on a 5 point scale. The higher the total score, the higher the degree of fatigue is. The Chinese version of MFI has been applied to patients with high reliability and validity [8, 38]. The Cronbach ’s α value of the MFI was 0.91 in the current study.

### Measurement of PSS

The Multidimensional Scale of Perceived Social Support (MSPSS) was utilized to evaluate PSS [39]. The MSPSS consists of 12 items and 3 subscales with 4 items each concerning family support, friend support and support from others (such as relatives, colleagues and community leaders). The score of each item is given on a 7-point Likert scale, ranging from (1) ‘very strongly disagree' to (7) ‘very strongly agree'. The total scale score ranges from 12 to 84. The higher the total score, the higher the extent of social support perceived by the patient. High reliability and validity have been confirmed among various Chinese patients[40, 41]. The Cronbach ’s α value of the MSPSS was 0.95 in the current study.

### Measurement of hope

The Herth Hope Index (HHI) is a simplified psychometrical instrument to measure the extent of a patient’s hopefulness [42]. The HHI consists of 12 items and 3 subscales with 4 items each: temporality and future, positive readiness and expectancy, and interconnectedness. The score of each item is graded with a 4-point Likert scale, ranging from (1) ‘very strongly disagree' to (4) ‘very strongly agree'. Each item is summed to yield a total score ranging from 12 to 48. The higher the total score, the greater the extent of hopefulness. The Chinese version of HHS has been demonstrated to have good validity and reliabilityand be suitable for various Chinese patients [18, 43]. In this research, the Cronbach ’s α value of the HHI was 0.82.

### Measurement of optimism

The Life Orientation Test Revised (LOT-R) is the most widely applied test for measuring optimism [44], with high degree of reliability and validity when used with various Chinese patients [18, 41]. The LOT-R comprises 10 items, including 6 scored items and 4 filler items. Among the 6 meaningful items, 3 are optimistic statements indicating positive expectations of future life, and 3 statements are pessimistic ones framing negative expectations. Each item is employed on a 5-point Likert scale, ranging from (1) ‘very strongly disagree' to (5) ‘very strongly agree '. A total score is calculated as the final outcomes ranging from 6 to 30. The higher the score, the more optimistic the patient is understood to be. In our research, the Cronbach ’s α value of the LOT-R was 0.86.

### Measurement of general self-efficacy

General self-efficacy was evaluated by the Chinese version of General Self-Efficacy Scale(GSES) [45], which is widely used among patients in China [43, 46]. The GSES comprises of 10 items with scores given on a 4-point Likert scale, ranging from (1) ‘completely incorrect' to (4) ‘completely correct’. The summed up total score of the GSES ranges from 10–40, with higher scores indicating higher patient confidence. With high validity and reliability, the Cronbach ’s α value of the GSES was 0.80 in the current study.

### Measurement of resilience

Resilience was evaluated by the Chinese version of the Ego-Resiliency Scale (ERS) [47]. The ERS consists of 14 items. The score of each item is evaluated on a 4-point Likert scale, ranging from (1) ‘does not apply at all' to (4) ‘completely apply '. The total scale score ranges from 14 to 56, with higher scores indicating higher levels of resilience and swifter recovery from emergencies. The ERS fits for different Chinese populations with high validity and reliability [48, 49], with the a Cronbach ’s α value of 0.88 in the current study.

### Demographic characteristics

Demographic variables included age, gender, marital status, educational level, employment and monthly income per capita. Marital status was sorted into 2 groups: married/cohabited and single/divorced/widowed/separated. Educational level was classified as junior high school or below, senior high school and junior college or above. Employment was sorted into 3 groups as unemployment, part-time and full-time. Clarified as 3 groups also, monthly income per capita was also classified into three groups of <3,000, 3,000–3,999 and ≥4,000 Yuan (RMB).

### Clinical variables

Clinical data included family history of RA, anemia, compliance of taking medicine, presence of other chronic comorbidities, duration of RA, EMS, TJC, SJC, CRP and ESR. Family history, anemia and other chronic comorbidities were grouped as either yes or no. Compliance of taking medicine was sorted into 3 groups: never, sometimes and always. Duration of suffering RA was classified as: 1, 2–5, 6–10 and >10 years. EMS was categorized as: <0.5, 0.5–1 and >1 hour. The disease activity was evaluated by ESR and DAS28-CRP. Based on CRP, TJC and SJC, DAS28-CRP was calculated according to the formula:

DAS28-CRP = [0.56*sqrt(TJC28)+0.28*sqrt(SJC28)+0.36*ln(CRP+1)]*1.10+ 1.15 and was sorted into 4 groups: clinical remission ≤2.6, low level of disease activity 2.6–3.2, moderate level 3.2–5.1 and high level >5.1.

### Statistical analysis

Statistical analysis was carried out by SPSS 13.0 software and P<0.05 (two-tailed) was viewed as statistically significant. Descriptive statistics were used to process the demographic, clinical and psychological variables manifested with number (n), percentage (%), mean and standard deviation (SD). Independent sample t-test and one-way analysis of variance (ANOVA) were utilized to evaluate the variations in fatigue concerning the demographics and clinical variables. Pearson’s correlation was applied to evaluate correlations among continuous variables.

Hierarchical regression analysis was applied to explore the associations of PSS, hope, optimism, general self-efficacy, and resilience with fatigue along with the moderating roles of internal psychological variables on the PSS-fatigue association involved in demographic and clinical variables, of which variables with P<0.25 as indicated by univariate analysis were added to the regression model besides age and gender. Demographic and clinical variables were included in Block 1, with PSS in Block 2, and hope, optimism, general self-efficacy and resilience in Block 3. Finally, the products of PSS with internal psychological constructs were employed in Block 4. If the interaction effect was statistically significant, simple slope analyses were conducted to visualize the interaction term.

Asymptotic and resampling strategies were employed to explore whether hope, optimism, general self-efficacy and resilience mediated the PSS-fatigue association. A bias-corrected and accelerated 95% confidence interval (BCa 95% CI) for each mediation was conducted with the bootstrap estimate on a basis of 5,000 bootstrap samples, in which exclusion of 0 implied that the variables indeed contributed as mediating factors to take effect.

Variables in the models were standardized before regression analysis to eliminate differences among scale scores. Multicollinearity was verified involving variance inflation factor (VIF).

## Results

### Descriptive statistics

The processed data of demographic and clinical characteristics and group differences among the different categories are displayed in Table 1.

**Table 1:** Demographic and clinical characteristics and group differences of fatigue.

Variables	*n*	%	Fatigue		*F*/*t* value	*P* value
			Mean	SD		
**Total**	**305**		**56.88**	**9.50**		
**Demographic variables**						
**Gender**					0.50	0.619
Men	69	22.62	56.38	9.93		
Women	236	77.38	57.03	9.39		
**Marital status**					2.97**	0.003
Married/cohabited	267	87.54	56.28	9.27		
Single/divorced/widowed/separated	38	12.46	61.11	10.14		
**Educational level**					0.09	0.915
Junior high school or below	105	34.43	56.56	9.37		
Senior high school	135	44.26	57.04	10.04		
Junior college or above	65	21.31	57.06	8.63		
**Employment**					3.07*	0.048
Unemployment	189	61.97	57.70	9.52		
Part-time	76	24.92	56.53	9.35		
Full-time	40	13.11	53.68	9.18		
**Monthly income per capita (yuan)**					6.60**	0.002
<3000	111	36.39	54.34	8.29		
3000–3999	124	40.66	58.60	10.19		
≥4000	70	22.95	57.84	9.28		
**Clinical variables**						
**History**					0.79	0.430
Yes	8	2.62	54.25	7.61		
No	297	97.38	56.95	9.55		
**Anemia**					0.77	0.440
Yes	125	41.0	57.38	9.09		
No	180	59.0	56.53	9.79		
**Duration (years)**					0.36	0.786
≤1	89	29.2	56.24	6.28		
2–5	84	27.5	57.48	5.55		
6–10	54	17.7	57.50	4.01		
>10	78	25.6	56.54	6.38		
**EMS (hours)**					3.53*	0.030
<0.5	100	32.8	57.44	9.66		
0.5–1	73	23.9	54.34	7.89		
>1	132	43.3	57.86	9.96		
**DAS28 CRP**					2.44	0.065
≤2.6	30	9.8	54.43	8.23		
2.6–3.2	28	9.2	56.11	9.18		
3.2–5.1	145	47.5	56.17	9.52		
>5.1	102	33.4	58.81	9.70		
**Other chronic comorbidities**					1.53	0.127
Yes	197	52.2	56.26	9.43		
No	108	47.8	58.00	9.57		
**Compliance**					0.24	0.790
Never	181	59.34	56.57	9.52		
Sometimes	121	39.67	57.33	9.50		
Always	3	0.98	57.33	11.59		

PSS: perceived social support; SD: standard deviation; ESR: erythrocyte sedimentation rate; DAS28 CRP: DAS28 C-reaction protein;

*P<0.05,

**P<0.01.

There were 3 times as many females as there were males, and there was no difference of fatigue with regard to gender. Among all patients, married/cohabited people accounted for 87.54 of patients, who suffered less severity of fatigue than the single/divorced/widowed/separated patients (t = 3.00, P<0.01). Only 21.31% held a junior college degree or above, and no distinction of fatigue was discovered among people with different educational status. Unemployed patients were the largest group in terms of job status with 61.97%, and employment was found to affect the severity of fatigue (F = 3.07, P<0.05). Differences in monthly income per capita exerted certain impacts on fatigue as well (F = 6.96, P<0.01).

There were no statistically significant differences discovered between the associations of fatigue with family history, anemia, duration of RA, DAS28-CRP and compliance of taking medicine. Patients whose duration of EMS was in a range of 0.5–1 hours suffered from the least severity of fatigue compared to other groups (F = 3.53, P<0.05).

### Correlations among continuous variables

The correlations among age, ESR, DAS28-CRP, PSS, positive psychological constructs and fatigue-the dependent variable are displayed in Table 2. The mean value of fatigue was 56.88 (SD = 9.50). The average age of RA patients was 57.80 (SD = 12.46). Age, ESR and DAS28-CRP were positively correlated with the independent variable carrying great significance. Meanwhile, PSS, hope, optimism, general self-efficacy and resilience were all significantly negatively correlated with fatigue.

**Table 2:** Correlations among continuous variables.

Variables	Mean(SD)	Min	Max	*r* (Fatigue)
**Age (years)**	57.8(12.46)	21	83	0.19**
**DAS28 CRP**	4.57(1.53)	1.56	8.13	0.17**
**ESR (mm/hour)**	42.05(22.99)	2	117	0.18**
**PSS**	64.07(9.58)	37	84	-0.80**
**Hope**	36.81(3.56)	24	46	-0.79**
**Optimism**	22.04(3.39)	16	29	-0.78**
**General self-efficacy**	18.27(3.62)	10	40	-0.57**
**Resilience**	34.16(5.46)	23	56	-0.69**

SD: standard deviation; Min: minimum; Max: maximum; *r* (Fatigue): the correlation with fatigue; PSS: perceived social support; ESR: erythrocyte sedimentation rate; DAS28 CRP: DAS28 C-reaction protein;

**P<0.01.

### Hierarchical regression analysis

**Fig 1:**
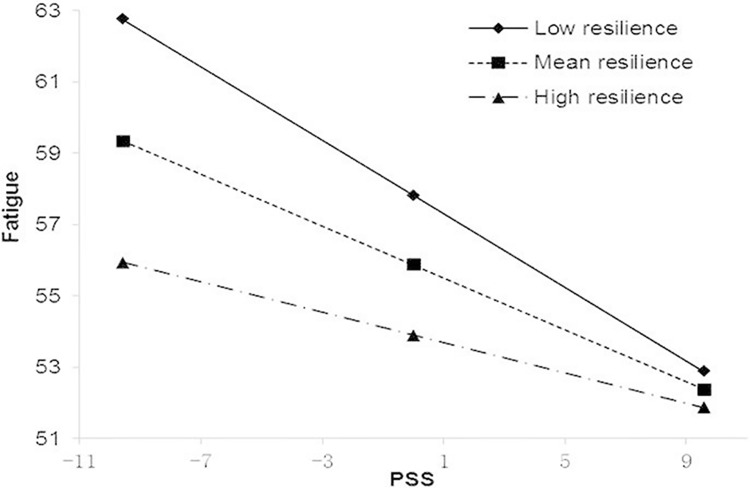
Moderating effect of resilience on PSS-fatigue association.

The results of hierarchical regression analysis are displayed in Table 3. In Block 1, age and other chronic comorbidities were negatively associated with fatigue, whereas the monthly income per capita and DAS28-CRP had a positive association. However, in Block 2 among the demographic and clinical variables, only DAS28-CRP was found to have a positive association with fatigue (B = 0.90, β = 0.15, P<0.01), and similar results were shown both in Blocks 3 and 4. Meanwhile, as shown in Block 2, there was a significant negative correspondence between PSS and fatigue as hypothesized (B = -0.76, β = -0.76,P<0.01) and PSS accounted for 49.7% of the variance of fatigue. In Block 3, hope, optimism and resilience were all negatively associated with fatigue while general self-efficacy was not (hope: B = -0.52, β = -0.20 P<0.01; optimism: B = -0.70,β = -0.25 P<0.01; resilience: B = -0.31, β = -0.18 P<0.01), together accounting for additional 6.6% of the variance playing a predictive role in fatigue. In Block 4, apart from the negative association of PSS, hope, optimism and resilience with fatigue, the product of PSS and resilience was positively associated with fatigue (B = 0.03, β = 0.13,P<0.01), accounting for additional 1.4% of the variance of fatigue. Simple slope analysis revealed that when resilience is lower, the association between PSS and fatigue becomes stronger. In other words, the associations between PSS and fatigue were gradually reduced in the low (1 SD below the mean, B = -0.52, β = -0.52, P<0.01), mean (B = -0.36, β = -0.37, P<0.01) and high (1 SD above the mean, B = -0.21, β = -0.21, P<0.01) groups of resilience. The interaction was visualized in Fig 1.

**Table 3:** Associations of PSS, positive psychological constructs with fatigue and moderating roles of positive psychological constructs.

Variables	Block 1			Block 2			Block 3			Block 4		
	B	SE	β	B	SE	β	B	SE	β	B	SE	β
**Gender**	1.20	1.27	0.05	0.85	0.79	0.04	0.45	0.71	0.02	0.18	0.70	0.01
**Age (years)**	0.13	0.06	0.18*	0.05	0.04	0.07	0.05	0.03	0.06	0.04	0.03	0.06
**Marital status**	-4.40	1.57	-0.14**	-1.20	0.99	-0.04	-0.35	0.90	-0.01	-0.06	0.88	0.00
**Employment**												
** Employment 1**	1.61	1.45	0.07	0.54	0.90	0.02	0.66	0.82	0.03	0.35	0.80	0.02
** Employment 2**	-0.75	1.94	-0.04	-0.25	1.21	-0.02	0.12	1.09	0.00	0.28	1.07	0.01
**Monthly income per capita (yuan)**												
** Monthly income per capita 1**	4.27	1.20	0.22**	0.64	0.77	0.03	0.20	0.69	0.01	-0.20	0.68	-0.01
** Monthly income per capita 2**	5.02	1.41	0.23**	-0.04	0.91	0.00	-0.13	0.81	0.00	-0.65	0.81	-0.03
**EMS (hours)**												
** EMS 1**	-3.76	1.42	-0.16*	-1.52	0.89	-0.07	-0.92	0.80	-0.04	-0.77	0.80	-0.03
** EMS 2**	-0.78	1.30	-0.03	-0.25	0.81	-0.01	-0.31	0.73	-0.01	-0.35	0.71	-0.02
**DAS28 CRP**	0.95*	0.41	0.15*	0.90	0.25	0.15**	0.84	0.23	0.14**	0.84	0.23	0.14
**ESR**	0.04	0.03	0.09	0.02	0.02	0.04	0.01	0.01	0.02	0.01	0.01	0.02
**Other chronic comorbidities**	-2.73	1.15	-0.14*	-0.77	0.72	-0.04	-0.05	0.66	-0.01	-0.08	0.65	-0.01
**PSS**				-0.76	0.04	-0.76**	-0.34	0.06	-0.34**	-0.36	0.06	-0.37**
**Hope**							-0.52	0.17	-0.20**	-0.44	0.17	-0.16*
**Optimism**							-0.70	0.16	-0.25**	-0.58	0.17	-0.21**
**General Self-efficacy**							0.18	0.12	0.07	0.12	0.13	0.04
**Resilience**							-0.31	0.09	-0.18**	-0.36	0.09	-0.20**
PSS*Hope										-0.02	0.01	-0.06
PSS*Optimism										-0.02	0.02	-0.04
PSS*General self-efficacy										0.00	0.02	0.00
PSS*Resilience										0.03	0.01	0.13**
**F**	5.51**			47.76**			49.87**			42.83**		
**df**	(12, 292)			(13, 291)			(17, 287)			(17, 287)		
**R2**	0.19			0.68			0.75			0.76		
**adjusted R2**	0.15			0.67			0.73			0.74		
**ΔR2**	0.19**			0.50**			0.07**			0.01**		

PSS: Perceived social support; Gender: men vs. women; Marital status: single/divorced/widowed/separated vs. married/cohabited; Employment 1: part-time vs. unemployment; Employment 2: full-time vs. unemployment; Monthly income per capita 1: 3000–3999 vs. <3000; Monthly income per capita 2: ≥4000 vs. <3000; EMS 1: 0.5–1 vs. <0.5; EMS 2: >1 vs. <0.5; Other chronic comorbiditites: no vs. yes; ESR: erythrocyte sedimentation rate; DAS28 CRP: DAS28 C-reaction protein;

*P<0.05,

**P<0.01.

Furthermore, the absolute value of β coefficient of PSS in Block3 (β = -0.34) was much smaller than that in Block2(β = -0.76) suggesting a lesser effect of PSS exerted on fatigue, indicating that the 4 positive psychological constructs could indirectly affect the association of PSS with fatigue as potential mediators.

Hope, optimism and resilience seem to act as mediators in the association between PSS and fatigue. The associations between PSS and hope, optimism, general self-efficacy along with resilience are referred to as ‘a’, the associations between hope, optimism, general self-efficacy along with resilience with fatigue after controlling for PSS are referred to as ‘b’, the value of their mediating effects, referred to as ‘a*b’ products and ‘a, b, a*b’ products along with their BCa 95% CI are displayed in Table 4.

**Table 4:** Mediating roles of positive psychological constructs on the PSS-fatigue association.

Mediators	a	b	a*b value	BCa95%CI	c'	c
**Hope**	0.80**	-0.20**	-0.16	(-0.27, -0.03)	-0.34	-0.76
**Optimism**	0.78**	-0.25**	-0.20	(-0.30, -0.10)		
**General self-efficacy**	0.67**	0.07	0.05	(-0.04, 0.13)		
**Resilience**	0.66**	-0.18**	-0.12	(-0.21, -0.04)		

PSS: Perceived social support; BCa 95% CI: Bias-corrected and accelerated 95% confidence interval; a: PSS → Hope/Optimism/ General Self-efficacy/Resilience; b: Hope/Optimism/ General Self-efficacy/Resilience → Fatigue; a*b: Product of a and b; c: PSS → Fatigue; c’: PSS → Hope, Optimism, General Self-efficacy and Resilience→ Fatigue. Gender, age, marital status, employment, monthly income per capita, duration of early morning stiffness (EMS), DAS28 CRP, erythrocyte sedimentation rate (ESR), and other chronic comorbidities were covariates.

**P<0.01.

Hope, optimism and resilience not only had significant and positive associations with PSS, but also exerted direct effects on fatigue after controlling for predictor variables, indicating that hope, optimism and resilience could act as mediators affecting the association between PSS and symptoms of fatigue (hope: a*b = -0.16, BCa 95%CI: -0.27,-0.03; optimism: a*b = -0.20, BCa 95%CI: -0.30, -0.10; resilience: a*b = -0.12, BCa 95%CI: -0.21,-0.04). Although general self-efficacy was positively associated with PSS, it did not have significant influence. Thus, general self-efficacy did not seem to mediate the association of PSS with fatigue.

Using the formula: (a*b)/total effect, the proportion of indirect effects of PSS on fatigue through mediators accounting for total effects could be calculated. With respect to fatigue, the proportions of mediating roles of hope, optimism, and resilience were 20.7%, 25.7% and 15.2% respectively.

## Discussion

In our research, the mean score of the MFI was much higher than that of the general population [50], demonstrating that fatigue is indeed serious in RA. Furthermore, Our results were corresponded with previous research of RA patients such as that in the Netherlands and Denmark [51, 52]. Similar research has never been conducted in China, sot no comparison may presently be made in the same population. Although the current study lacks without control groups of patients suffering from other diseases, the scores above are at a medium-high level with regard to related researches carried out among other clinical populations [53–56]. PSS had a significantly negative association with fatigue, accounting for almost half of the variance concerning its predicting role, indicating that less severity of fatigue might be experienced among RA patients when acquiring more positive assistance from others. Compared with the US and Egypt, RA patients in the UK and the Netherlands seemed to be dealing with their fatigue by themselves without seeking help from others, thus suffering from greater severity of symptoms [5, 57–59]. A Chinese study reported that social support, as a direct or indirect factor, played an important predicting role in health-related quality of life both physically and psychologically[8]. But some researches have reported that patients with RA always lack adequate social support [15, 16], probably because RA is incurable with a prolonged course, causing even deformity and physical disability in the final stage. Furthermore, most patients suffering RA are usually involved in restricted social activities, contributing to less support from the society [15, 57]. Therefore, social support may be quite relevant to RA-related fatigue and could probably relieve it to a large extent. The current study has shown that, hope, optimism and resilience, contributed as mediators and affected the PSS-fatigue association by accounting for a proportion of the variance in predicting fatigue, indicating that RA patients might experience less severity of fatigue when they had higher levels of hope, optimism and resilience. However, research and evidence regarding their impact among patients with RA is quite rare.

RA patients burdened by a prolonged course of disease, achieve hope not by relying on any exact target but by attaching importance to present or future possibilities, as well as themselves or others they viewed to be important, which is different from the usual “goal orientation” [60]. When going through declining functioning even physical disability in the progress of the disease, it is hope that contributed to the intrinsic strength that beings patients the courage to confront reality with a positive attitude. Meanwhile, hope can promote patients’ adaption to the difficult condition, rather than extricating themselves from it. Therefore, higher level of hope could probably relieve fatigue among RA patients.

Apart from its contribution as a personality trait, optimism also acts as an explanatory pattern bound up with positive prospects [61], exerting desirable influences on psychological health. Optimism exerts considerable impacts on accommodation with chronic disease, such as RA, making it great beneficial to the physical functioning among patients in a direct or indirect way [62]. Further, RA patients with a higher level of optimism achieved a better emotional status [63, 64], indicating that a positive and optimistic mood can alleviate fatigue to a certain extent. Higher level of optimism resulted in less pain, thus improving the wellbeing among RA patients [63]. But researches demonstrated that RA patients did have worse expectations of their fatigue symptoms [65]. As a result, optimism probably has an impact on RA-related fatigue.It was shown that resilience was expected to predict and evaluate fatigue symptom among cancer patients [36]. Although resilience was studied only in a few studies of RA, a consensus was reached that higher level of resilience was beneficial for RA patients [66, 67] and a study reported that resilience was positively associated with PSS [68]. In our research, resilience not only acted as a mediator by playing a predictive role, but it also positively moderated on the PSS-fatigue association, indicating that with higher levels of resilience, the association between PSS and fatigue might be more close among RA patients. Therefore, improving resilience could probably alleviate fatigue among RA patients.

Although negatively correlated to fatigue in univariate analysis, self-efficacy did not have statistical significance in the regression analysis. However, previous researches have reported that patients with higher levels of self-efficacy suffered from less fatigue severity in RA [8, 17]. This difference maybe because the effect of self-efficacy was covered by other psychological variables. However, another study also indicated that the association between self-efficacy and fatigue was not confirmed by multivariate analysis [17]. Therefore, further research should be conducted to explore its exact effect in RA-related fatigue.

The current study provided the preliminary possibility of building a core-positive high-order psychological construct to alleviate fatigue in RA patients by utilizing the individual positive construct of hope, optimism and resilience. Therefore, several clinical implications might be concluded according to this study. First, fatigue is a serious among RA patients, and much more attention should be paid to detecting and treating its symptoms by Chinese rheumatologists. Besides, social support may be taken into account seriously with regard to the alleviation of RA-related fatigue. In addition, a whole new perspective could emerge on the application of a model to improve positive psychological resources and relieve fatigue in RA patients by utilizing the protective effects of social support, hope, optimism and resilience. As the present study has demonstrated, social support, hope, optimism and resilience all probably have positive impacts on alleviating fatigue, so non-pharmacological treatments with regard to RA-related fatigue may focus on social support and positive psychological interventions mainly targeting hope, optimism and resilience. As for social support, family, friends and society should pay more attention to these patients, and doctors should show more solicitude as well. Encouragements from doctors can enhance patients confidence during treatments and improve their compliance, thereby relieving fatigue symptoms remarkably [4, 6]. Online interactions aimed at acquiring more social support have also been shown to be beneficial to RA patients, which can also be recommended [12]. As for hope, optimism and resilience, these positive psychological constructs may keep patients maintain a positive and optimistic attitude towards confronting reality and coordinating with doctors, thus managing fatigue symptoms successfully. Therefore, adequate social support and positive psychological interventions targeting hope, optimism and resilience should probably be attached great importance to alleviate fatigue symptoms among RA patients. However, there are still several limitations in our research. First, self-reporting measures were applied to evaluate patients’ conditions and some of them fulfilled the questionnaires with the help of others due to illiteracy, which might lead to recall and reporting bias affecting the associations among variables. Therefore, efficacious control measures were required, such as the guarantee of anonymity, measuring instruments with high reliability and validity and emphasizing to patients there was no right or wrong answer. Second, our research was conducted in northeast China at various multicenters, but whether the results may be generalized to other populations with different cultural backgrounds requires further study. Third, the cross-sectional method applied in our research only provided the general condition of patients at the exact time point of investigation, thus restricting the evaluation of casual links among the variables. Therefore, prospective longitudinal studies need to be studied to further verify our conclusions.

## Conclusions

Fatigue is a severe symptom among RA patients. Only resilience was shown to probably positively moderates the PSS-fatigue association. Hope, optimism and resilience may act as partial mediators in the association between PSS and fatigue. PSS, hope, optimism and resilience may contribute as effective recourses to alleviate fatigue, with PSS showing the greatest possible effect. Adequate social support and positive psychological interventions targeted at hope, optimism and resilience should probably be attached great importance to alleviate fatigue symptom among RA patients.

## Supporting information

S1 File STROBE_checklist_v4_combined_PlosMedicine. (DOCX)

## References

[1] Taylor PC, Moore A, Vasilescu R, Alvir J, Tarallo M. A structured literature review of the burden of illness and unmet needs in patients with rheumatoid arthritis: a current perspective. Rheumatology international. 2016;36(5):685–95. Epub 2016/01/10. 10.1007/s00296-015-3415-x 26746843 PMC4839053

[2] Xiang YJ, Dai SM. Prevalence of rheumatic diseases and disability in China. Rheumatology international. 2009;29(5):481–90. Epub 2008/12/11. 10.1007/s00296-008-0809-z 19066899

[3] Feldthusen C, Dean E, Forsblad-d'Elia H, Mannerkorpi K. Effects of Person-Centered Physical Therapy on Fatigue-Related Variables in Persons With Rheumatoid Arthritis: A Randomized Controlled Trial. Archives of physical medicine and rehabilitation. 2016;97(1):26–36. Epub 2015/10/21. 10.1016/j.apmr.2015.09.022 26482574

[4] Mayoux-Benhamou MA. Fatigue and rheumatoid arthritis. Annales de readaptation et de medecine physique: revue scientifique de la Societe francaise de reeducation fonctionnelle de readaptation et de medecine physique. 2006;49(6):301–4, 85–8. Epub 2006/06/03. 10.1016/j.annrmp.2006.04.011 16740333

[5] Repping-Wuts H, Uitterhoeve R, van Riel P, van Achterberg T. Fatigue as experienced by patients with rheumatoid arthritis (RA): a qualitative study. International journal of nursing studies. 2008;45(7):995–1002. Epub 2007/07/31. 10.1016/j.ijnurstu.2007.06.007 17662291

[6] van Steenbergen HW, Tsonaka R, Huizinga TW, Boonen A, van der Helm-van Mil AH. Fatigue in rheumatoid arthritis; a persistent problem: a large longitudinal study. RMD open. 2015;1(1):e000041 Epub 2015/10/29. 10.1136/rmdopen-2014-000041 26509063 PMC4612698

[7] Nikolaus S, Bode C, Taal E, van de Laar MA. Fatigue and factors related to fatigue in rheumatoid arthritis: a systematic review. Arthritis care & research. 2013;65(7):1128–46. Epub 2013/01/22. 23335492 10.1002/acr.21949

[8] Gong G, Mao J. Health-Related Quality of Life Among Chinese Patients With Rheumatoid Arthritis: The Predictive Roles of Fatigue, Functional Disability, Self-Efficacy, and Social Support. Nursing research. 2016;65(1):55–67. Epub 2015/12/15. 10.1097/NNR.0000000000000137 26657481

[9] Hewlett S, Dures E, Almeida C. Measures of fatigue: Bristol Rheumatoid Arthritis Fatigue Multi-Dimensional Questionnaire (BRAF MDQ), Bristol Rheumatoid Arthritis Fatigue Numerical Rating Scales (BRAF NRS) for severity, effect, and coping, Chalder Fatigue Questionnaire (CFQ), Checklist Individual Strength (CIS20R and CIS8R), Fatigue Severity Scale (FSS), Functional Assessment Chronic Illness Therapy (Fatigue) (FACIT-F), Multi-Dimensional Assessment of Fatigue (MAF), Multi-Dimensional Fatigue Inventory (MFI), Pediatric Quality Of Life (PedsQL) Multi-Dimensional Fatigue Scale, Profile of Fatigue (ProF), Short Form 36 Vitality Subscale (SF-36 VT), and Visual Analog Scales (VAS). Arthritis care & research. 2011;63 Suppl 11:S263–86. Epub 2012/05/25. 22588750 10.1002/acr.20579

[10] van Hoogmoed D, Fransen J, Repping-Wuts H, Spee L, Bleijenberg G, van Riel PL. The effect of anti-TNF-alpha vs. DMARDs on fatigue in rheumatoid arthritis patients. Scandinavian journal of rheumatology. 2013;42(1):15–9. Epub 2012/09/21. 10.3109/03009742.2012.709878 22992002

[11] van Hoogmoed D, Fransen J, Bleijenberg G, van Riel P. Physical and psychosocial correlates of severe fatigue in rheumatoid arthritis. Rheumatology (Oxford, England). 2010;49(7):1294–302. Epub 2010/04/01. 10.1093/rheumatology/keq043 20353956

[12] Kostova Z, Caiata-Zufferey M, Schulz PJ. Can social support work virtually? Evaluation of rheumatoid arthritis patients' experiences with an interactive online tool. Pain research & management. 2015;20(4):199–209. Epub 2015/08/08. 26252664 10.1155/2015/497512 PMC4532206

[13] Lakey B, Orehek E, Hain KL, Van Vleet M. Enacted support's links to negative affect and perceived support are more consistent with theory when social influences are isolated from trait influences. Personality & social psychology bulletin. 2010;36(1):132–42. Epub 2009/10/31. 19875827 10.1177/0146167209349375

[14] Smith TW, Ruiz JM, Uchino BN. Mental activation of supportive ties, hostility, and cardiovascular reactivity to laboratory stress in young men and women. Health psychology: official journal of the Division of Health Psychology, American Psychological Association. 2004;23(5):476–85. Epub 2004/09/16. 10.1037/0278-6133.23.5.476 15367067

[15] McInnes IB, Combe B, Burmester G. Understanding the patient perspective—results of the Rheumatoid Arthritis: Insights, Strategies & Expectations (RAISE) patient needs survey. Clinical and experimental rheumatology. 2013;31(3):350–7. Epub 2013/02/15. 23406685

[16] Kostova Z, Caiata-Zufferey M, Schulz PJ. The impact of social support on the acceptance process among RA patients: a qualitative study. Psychology & health. 2014;29(11):1283–302. Epub 2014/05/21. 24841727 10.1080/08870446.2014.925895

[17] Matcham F, Ali S, Hotopf M, Chalder T. Psychological correlates of fatigue in rheumatoid arthritis: a systematic review. Clinical psychology review. 2015;39:16–29. Epub 2015/04/29. 10.1016/j.cpr.2015.03.004 25912978

[18] Liu L, Yang YL, Wang ZY, Wu H, Wang Y, Wang L. Prevalence and Positive Correlates of Posttraumatic Stress Disorder Symptoms among Chinese Patients with Hematological Malignancies: A Cross-Sectional Study. PloS one. 2015;10(12):e0145103 Epub 2015/12/17. 10.1371/journal.pone.0145103 26669841 PMC4679613

[19] Dufault K, Martocchio BC. Symposium on compassionate care and the dying experience. Hope: its spheres and dimensions. The Nursing clinics of North America. 1985;20(2):379–91. Epub 1985/06/01. 3846980

[20] Irving LM, Telfer L, Blake DD. Hope, coping, and social support in combat-related posttraumatic stress disorder. Journal of traumatic stress. 1997;10(3):465–79. Epub 1997/07/01. 9246653 10.1023/a:1024897406135

[21] Heidari M, Ghodusi M. The Relationship between Body Esteem and Hope and Mental Health in Breast Cancer Patients after Mastectomy. Indian journal of palliative care. 2015;21(2):198–202. Epub 2015/05/27. 10.4103/0973-1075.156500 26009674 PMC4441182

[22] Hirsch JK, Sirois FM. Hope and fatigue in chronic illness: The role of perceived stress. Journal of health psychology. 2016;21(4):451–6. Epub 2014/03/29. 10.1177/1359105314527142 24677432

[23] Kreis S, Molto A, Bailly F, Dadoun S, Fabre S, Rein C, et al Relationship between optimism and quality of life in patients with two chronic rheumatic diseases: axial spondyloarthritis and chronic low back pain: a cross sectional study of 288 patients. Health and quality of life outcomes. 2015;13:78 Epub 2015/07/08. 10.1186/s12955-015-0268-7 26149393 PMC4491882

[24] Scheier MF, Carver CS. Optimism, coping, and health: assessment and implications of generalized outcome expectancies. Health psychology: official journal of the Division of Health Psychology, American Psychological Association. 1985;4(3):219–47. Epub 1985/01/01. 10.1037//0278-6133.4.3.219 4029106

[25] Carver CS, Scheier MF, Segerstrom SC. Optimism. Clinical psychology review. 2010;30(7):879–89. Epub 2010/02/23. 10.1016/j.cpr.2010.01.006 20170998 PMC4161121

[26] Avvenuti G, Baiardini I, Giardini A. Optimism's Explicative Role for Chronic Diseases. Frontiers in psychology. 2016;7:295 Epub 2016/03/15. 10.3389/fpsyg.2016.00295 26973582 PMC4773598

[27] Luszczynska A, Scholz U, Schwarzer R. The general self-efficacy scale: multicultural validation studies. The Journal of psychology. 2005;139(5):439–57. Epub 2005/11/16. 10.3200/JRLP.139.5.439-457 16285214

[28] Schwarzer R, Born A. Optimistic self-beliefs: Assessment of general perceived self-efficacy in thirteen cultures. World Psychology. 1997;3(1–2):177–90.

[29] Schwarzer R, Born A, Iwawaki S, Lee Y-M. The assessment of optimistic self-beliefs: Comparison of the Chinese, Indonesian, Japanese, and Korean versions of the General Self-Efficacy scale. Psychologia: An International Journal of Psychology in the Orient. 1997.

[30] Shrivastava A, Desousa A. Resilience: A psychobiological construct for psychiatric disorders. Indian journal of psychiatry. 2016;58(1):38–43. Epub 2016/03/18. 10.4103/0019-5545.174365 26985103 PMC4776579

[31] Lawford J, Eiser C. Exploring links between the concepts of Quality of Life and resilience. Pediatric rehabilitation. 2001;4(4):209–16. Epub 2002/08/06. 10.1080/13638490210124024 12160361

[32] Yi JP, Smith RE, Vitaliano PP. Stress-resilience, illness, and coping: a person-focused investigation of young women athletes. Journal of behavioral medicine. 2005;28(3):257–65. Epub 2005/07/15. 10.1007/s10865-005-4662-1 16015460

[33] Bronfenbrenner U, Ceci SJ. Nature-nurture reconceptualized in developmental perspective: a bioecological model. Psychological review. 1994;101(4):568–86. Epub 1994/10/01. 7984707 10.1037/0033-295x.101.4.568

[34] Lim JW, Shon EJ, Paek M, Daly B. The dyadic effects of coping and resilience on psychological distress for cancer survivor couples. Supportive care in cancer: official journal of the Multinational Association of Supportive Care in Cancer. 2014;22(12):3209–17. Epub 2014/07/06. 24993394 10.1007/s00520-014-2334-9 PMC4221537

[35] Losoi H, Waljas M, Turunen S, Brander A, Helminen M, Luoto TM, et al Resilience is associated with fatigue after mild traumatic brain injury. The Journal of head trauma rehabilitation. 2015;30(3):E24–32. Epub 2014/05/21. 10.1097/HTR.0000000000000055 24842587

[36] Strauss B, Brix C, Fischer S, Leppert K, Fuller J, Roehrig B, et al The influence of resilience on fatigue in cancer patients undergoing radiation therapy (RT). Journal of cancer research and clinical oncology. 2007;133(8):511–8. Epub 2007/06/20. 10.1007/s00432-007-0195-z 17576595 PMC12160912

[37] Smets EM, Garssen B, Bonke B, De Haes JC. The Multidimensional Fatigue Inventory (MFI) psychometric qualities of an instrument to assess fatigue. Journal of psychosomatic research. 1995;39(3):315–25. Epub 1995/04/01. 7636775 10.1016/0022-3999(94)00125-o

[38] Tian J, Hong JS. Validation of the Chinese version of Multidimensional Fatigue Inventory-20 in Chinese patients with cancer. Supportive care in cancer: official journal of the Multinational Association of Supportive Care in Cancer. 2012;20(10):2379–83. Epub 2011/12/27. 22198167 10.1007/s00520-011-1357-8

[39] Zimet GD, Powell SS, Farley GK, Werkman S, Berkoff KA. Psychometric characteristics of the Multidimensional Scale of Perceived Social Support. Journal of personality assessment. 1990;55(3–4):610–7. Epub 1990/01/01. 10.1080/00223891.1990.9674095 2280326

[40] Li MY, Yang YL, Liu L, Wang L. Effects of social support, hope and resilience on quality of life among Chinese bladder cancer patients: a cross-sectional study. Health and quality of life outcomes. 2016;14:73 Epub 2016/05/08. 10.1186/s12955-016-0481-z 27153944 PMC4859956

[41] Yang YL, Liu L, Li MY, Shi M, Wang L. Psychological Disorders and Psychosocial Resources of Patients with Newly Diagnosed Bladder and Kidney Cancer: A Cross-Sectional Study. PloS one. 2016;11(5):e0155607 Epub 2016/05/19. 10.1371/journal.pone.0155607 27191964 PMC4871582

[42] Herth K. Development and refinement of an instrument to measure hope. Scholarly inquiry for nursing practice. 1991;5(1):39–51; discussion 3–6. Epub 1991/01/01. 2063043

[43] Yang YL, Liu L, Wang XX, Wang Y, Wang L. Prevalence and associated positive psychological variables of depression and anxiety among Chinese cervical cancer patients: a cross-sectional study. PloS one. 2014;9(4):e94804 Epub 2014/04/12. 10.1371/journal.pone.0094804 24722558 PMC3983270

[44] Scheier MF, Carver CS, Bridges MW. Distinguishing optimism from neuroticism (and trait anxiety, self-mastery, and self-esteem): a reevaluation of the Life Orientation Test. Journal of personality and social psychology. 1994;67(6):1063–78. Epub 1994/12/01. 7815302 10.1037//0022-3514.67.6.1063

[45] Schwarzer R, Jerusalem M, In Weinman J. Measures in health psychology. Generalized Self-Efficacy Scale. 1995:35–7.

[46] Zhang JX, Schwarzer R. Measuring optimistic self-beliefs: A Chinese adaptation of the General Self-Efficacy Scale. Psychologia: An International Journal of Psychology in the Orient. 1995.

[47] Block J, Kremen AM. IQ and ego-resiliency: conceptual and empirical connections and separateness. Journal of personality and social psychology. 1996;70(2):349–61. Epub 1996/02/01. 8636887 10.1037//0022-3514.70.2.349

[48] Yang H, Guo W. Chinese version of the Responses to Positive Affect Questionnaire: testing the factor structure, reliability, and validity in a college student sample. Psychological reports. 2014;115(2):467–84. Epub 2014/09/23. 10.2466/08.21.PR0.115c22z8 25243367

[49] Zhai J, Huang Y, Gao X, Jiang H, Xu J. Post-trauma growth in a mainland Chinese population with chronic skin disease. International journal of dermatology. 2014;53(4):450–7. Epub 2014/03/22. 10.1111/j.1365-4632.2012.05734.x 24650075

[50] Schwarz R, Krauss O, Hinz A. Fatigue in the general population. Onkologie. 2003;26(2):140–4. Epub 2003/05/29. 12771522 10.1159/000069834

[51] Loppenthin K, Esbensen BA, Ostergaard M, Jennum P, Tolver A, Aadahl M, et al Physical activity and the association with fatigue and sleep in Danish patients with rheumatoid arthritis. Rheumatology international. 2015;35(10):1655–64. Epub 2015/05/08. 10.1007/s00296-015-3274-5 25947325

[52] Rupp I, Boshuizen HC, Jacobi CE, Dinant HJ, van den Bos GA. Impact of fatigue on health-related quality of life in rheumatoid arthritis. Arthritis and rheumatism. 2004;51(4):578–85. Epub 2004/08/31. 10.1002/art.20539 15334430

[53] Abd El-Kader SM, Al-Jiffri OH, Al-Shreef FM. Aerobic exercises alleviate symptoms of fatigue related to inflammatory cytokines in obese patients with type 2 diabetes. African health sciences. 2015;15(4):1142–8. Epub 2016/03/10. 10.4314/ahs.v15i4.13 26958015 PMC4765418

[54] Xiao C, Beitler JJ, Higgins KA, Conneely K, Dwivedi B, Felger J, et al Fatigue is associated with inflammation in patients with head and neck cancer before and after intensity-modulated radiation therapy. Brain, behavior, and immunity. 2016;52:145–52. Epub 2015/10/31. 10.1016/j.bbi.2015.10.016 26515035 PMC4867228

[55] Lund Rasmussen C, Klee Olsen M, Thit Johnsen A, Petersen MA, Lindholm H, Andersen L, et al Effects of melatonin on physical fatigue and other symptoms in patients with advanced cancer receiving palliative care: A double-blind placebo-controlled crossover trial. Cancer. 2015;121(20):3727–36. Epub 2015/07/17. 10.1002/cncr.29563 26178160

[56] Weissflog G, Brahler E, Leuteritz K, Barthel Y, Kuhnt S, Wiltink J, et al Does psychodynamic short-term psychotherapy for depressed breast cancer patients also improve fatigue? Results from a randomized controlled trial. Breast cancer research and treatment. 2015;152(3):581–8. Epub 2015/07/15. 10.1007/s10549-015-3494-0 26163828

[57] Mortada M, Abdul-Sattar A, Gossec L. Fatigue in Egyptian patients with rheumatic diseases: a qualitative study. Health and quality of life outcomes. 2015;13:134 Epub 2015/08/25. 10.1186/s12955-015-0304-7 26297320 PMC4546339

[58] Hewlett S, Cockshott Z, Byron M, Kitchen K, Tipler S, Pope D, et al Patients' perceptions of fatigue in rheumatoid arthritis: overwhelming, uncontrollable, ignored. Arthritis and rheumatism. 2005;53(5):697–702. Epub 2005/10/07. 10.1002/art.21450 16208668

[59] Tack BB. Fatigue in rheumatoid arthritis. Conditions, strategies, and consequences. Arthritis care and research: the official journal of the Arthritis Health Professions Association. 1990;3(2):65–70. Epub 1990/06/01. 2285744

[60] Kim DS, Kim HS, Schwartz-Barcott D, Zucker D. The nature of hope in hospitalized chronically ill patients. International journal of nursing studies. 2006;43(5):547–56. Epub 2005/09/06. 10.1016/j.ijnurstu.2005.07.010 16140301

[61] Hirsch JK, Conner KR. Dispositional and explanatory style optimism as potential moderators of the relationship between hopelessness and suicidal ideation. Suicide & life-threatening behavior. 2006;36(6):661–9. Epub 2007/01/26. 17250470 10.1521/suli.2006.36.6.661

[62] Fournier M, De Ridder D, Bensing J. Optimism and adaptation to chronic disease: The role of optimism in relation to self-care options of type 1 diabetes mellitus, rheumatoid arthritis and multiple sclerosis. British journal of health psychology. 2002;7(Part 4):409–32. Epub 2003/03/05. 10.1348/135910702320645390 12614494

[63] Treharne GJ, Kitas GD, Lyons AC, Booth DA. Well-being in rheumatoid arthritis: the effects of disease duration and psychosocial factors. Journal of health psychology. 2005;10(3):457–74. Epub 2005/04/29. 10.1177/1359105305051416 15857874

[64] McBain H, Shipley M, Newman S. The impact of appearance concerns on depression and anxiety in rheumatoid arthritis. Musculoskeletal care. 2013;11(1):19–30. Epub 2012/06/20. 10.1002/msc.1020 22711333

[65] Buitinga L, Braakman-Jansen LM, Taal E, van de Laar MA. Future expectations and worst-case future scenarios of patients with rheumatoid arthritis: a focus group study. Musculoskeletal care. 2012;10(4):240–7. Epub 2012/06/22. 10.1002/msc.1026 22718578

[66] Graninger M. [Behavioral training as additional therapy approach for rheumatoid arthritis]. Zeitschrift fur Rheumatologie. 2015;74(7):579–83. Epub 2015/09/04. 10.1007/s00393-014-1554-1 26334968

[67] Nasilowska-Barud A, Zuk M. [Chosen problems of mental functioning in patients with chronic systemic connective tissue diseases base on example of rheumatoid arthritis]. Wiadomosci lekarskie (Warsaw, Poland: 1960). 2015;68(3):279–83. Epub 2016/01/13. 26753214

[68] Kim J, Han JY, Shaw B, McTavish F, Gustafson D. The roles of social support and coping strategies in predicting breast cancer patients' emotional well-being: testing mediation and moderation models. Journal of health psychology. 2010;15(4):543–52. Epub 2010/05/13. 10.1177/1359105309355338 20460411 PMC3145334

