# Supplementary material for: Associations of perceived social support and positive psychological resources with fatigue symptom in patients with rheumatoid arthritis
Source: PLoS One. 2017 Mar 14;12(3):e0173293. doi: 10.1371/journal.pone.0173293 (PMC5349444; doi:10.1371/journal.pone.0173293)
Supplement: S1 File — (DOCX) [file pone.0173293.s001.docx]

STROBE Statement—checklist of items that should be included in reports of observational studies

|  | Item No. | Recommendation | Page  No. | Relevant text from manuscript | |
| --- | --- | --- | --- | --- | --- |
| **Title and abstract** | 1 | (*a*) Indicate the study’s design with a commonly used term in the title or the abstract | 2 | Cross-sectional study | |
|  |  | (*b*) Provide in the abstract an informative and balanced summary of what was done and what was found | 2-3 | Our study is a multi-center and cross-sectional study conducted in inpatients diagnosed with RA in northeast of China.  Various scales and statistical methods were completed to assess the associations of PSS, hope, optimism, general self-efficacy and resilience with fatigue. The moderating roles of these positive psychological constructs were tested by hierarchical linear regression, and asymptotic and resampling strategies were utilized to assess their mediating roles.  Fatigue is quite serious among RA patients. PSS, hope, optimism and resilience can contribute as effective recourses to alleviate fatigue, which PSS has the greatest effect. Resilience moderates the PSS-fatigue association positively. And hope, optimism and resilience could act as partial mediators in the association. | |
| Introduction | | | | |  |
| Background/rationale | 2 | Explain the scientific background and rationale for the investigation being reported | 3-6 | Fatigue is quite common among RA patients posing a substantial burden on the overall quality of life, thus decreasing patients’ general well-being.  Psychosocial strategies have gradually been considered a beneficial way to achieve alleviation of fatigue in recent years and the newly emerging positive psychology is under rapid development. But almost none researches have been conducted to evaluate the association among PSS, positive psychological constructs and fatigue in RA and how they interact closely with each other. More importance should be attached to PSS and psychological constructs to alleviate fatigue symptom in RA. | |
| Objectives | 3 | State specific objectives, including any prespecified hypotheses | 6 | 1. To assess the effect of PSS on fatigue among RA patients, adjusting for some related factors including demographic and clinical variables simultaneously.  2. To assess whether or not hope, optimism, general self-efficacy and resilience moderate or mediate the association between PSS and fatigue. | |
| Methods | | | |  | |
| Study design | 4 | Present key elements of study design early in the paper | 8-12 | Our study is a multi-center and cross-sectional study conducted in inpatients diagnosed with RA in northeast of China.  Various scales and statistical methods were completed to assess the associations of PSS, hope, optimism, general self-efficacy and resilience with fatigue. | |
| Setting | 5 | Describe the setting, locations, and relevant dates, including periods of recruitment, exposure, follow-up, and data collection | 7-8 | 1. Periods of recruitment: from 2014.12 to 2016.1  2. Exposure: Successive inpatients diagnosed with RA at Shengjing Hospital of China Medical University, Central Hospital of Benxi, General hospital of Fushun Mining Bureau and Sujiatun Central Hospital of Shenyang completed questionnaires.  3. There are no follow-ups in the study.  4. Data collection: Patients completed questionnaires, patients’ conditions were evaluated by physicians and the clinical data was collected from medical records. | |
| Participants | 6 | (*a*) *Cohort study*—Give the eligibility criteria, and the sources and methods of selection of participants. Describe methods of follow-up  *Case-control study*—Give the eligibility criteria, and the sources and methods of case ascertainment and control selection. Give the rationale for the choice of cases and controls  *Cross-sectional study*—Give the eligibility criteria, and the sources and methods of selection of participants | 7-8 | Cross-sectional study  1. The eligibility criteria:  Patients diagnosed with RA based on ACR/EULAR 2010 met the inclusion criteria followed: (1) ≥ 18 years; (2) had certain cognitive function; (3) were able to complete questionnaires consciously. The exclusion criteria included: (1) had psychiatric history and intellectual injury; (2) were taking psychotropic drugs; (3) were suffering from other severe illness simultaneously.  2. The sources of participants’ selection: Successive inpatients diagnosed with RA at Shengjing Hospital of China Medical University, Central Hospital of Benxi, General hospital of Fushun Mining Bureau and Sujiatun Central Hospital of Shenyang.  3. The methods of participants’ selection: All the eligible patients enrolled in the research voluntarily and anonymously understood the objectives and procedures of the research and signed the informed consent. Twelve respondents who refused to cooperate subsequently and eight respondents which missing information accounting for more than 30% were removed. | |
|  |  | (*b*) *Cohort study*—For matched studies, give matching criteria and number of exposed and unexposed  *Case-control study*—For matched studies, give matching criteria and the number of controls per case | - | Our study is a cross-sectional study. | |
| Variables | 7 | Clearly define all outcomes, exposures, predictors, potential confounders, and effect modifiers. Give diagnostic criteria, if applicable | 8-11 | 1.Outcomes: Fatigue  Predictors: Perceived Social Support  Potential confounders:  age, gender, marital status, educational level, employment and monthly income per capita, family history of RA, anemia, compliance of taking medicine, other chronic comorbidities, duration of suffering RA, duration of early morning stiffness (EMS), swollen joint counts (SJC) and tender joint counts (TJC), C-reaction protein (CRP) and erythrocyte sedimentation rate (ESR).  Potential effect modifiers: hope, optimism, general self-efficacy and resilience.  2. Diagnostic criteria: based on the 2010 ACR/EULAR RA criteria | |
| Data sources/ measurement | 8* | For each variable of interest, give sources of data and details of methods of assessment (measurement). Describe comparability of assessment methods if there is more than one group | 7-11 | 1. Source of data: Data were collected from the questionnaires completed by successive inpatients diagnosed with RA at Shengjing Hospital of China Medical University, Central Hospital of Benxi, General hospital of Fushun Mining Bureau and Sujiatun Central Hospital of Shenyang and their medical records.  2. Methods of measurements: The degree of Fatigue and psychological conducts were measured by various scales: The Multidimensional Fatigue Inventory; the Multidimensional Scale of Perceived Social Support; the Herth Hope Index; the Life Orientation Test Revised; the General Self-efficacy Scale and the Ego-Resiliency Scale. Variables in the models were standardized before regression analysis to eliminate differences among scale scores. The demographic and clinical variables were collected from the questionnaires completed by patients themselves and their medical records. | |
| Bias | 9 | Describe any efforts to address potential sources of bias | 7 | 1. We expanded the sample size as much as possible from four different hospitals.  2. All the eligible patients enrolled in the research voluntarily and anonymously understood the objectives and procedures of the research. After distributed, questionnaires were completed by the patient himself or with the help of others under the guidance of investigators. | |
| Study size | 10 | Explain how the study size was arrived at | 7-8 | Patients enrolled in the research were successive inpatients diagnosed with RA at Shengjing Hospital of China Medical University, Central Hospital of Benxi, General hospital of Fushun Mining Bureau and Sujiatun Central Hospital of Shenyang for one year. Among all the eligible patients enrolled, respondents who refused to cooperate and whose missing information accounting for more than 30% were removed. Finally, there were 305 subjects qualified for the research | |

Continued on next page

| Quantitative variables | 11 | Explain how quantitative variables were handled in the analyses. If applicable, describe which groupings were chosen and why | 11-12 | Age was classified as 3 groups: 40, 41-60 and >60 years old. Because the incidence of RA is high among the middle and old aged people.  Duration of early morning stiffness (EMS) was categorized as: <0.5, 0.5-1 and >1 hour. In 1987 RA diagnosed criteria, duration of EMS at least more than one hour is one of the criteria.  DAS28CRP was sorted into 4 groups: clinical remission ≤2.6, low level of disease activity 2.6-3.2, moderate level 3.2-5.1 and high level >5.1 according to the criteria.  Other continuous variables were treated just as continuous variables without any groupings. |
| --- | --- | --- | --- | --- |
| Statistical methods | 12 | (*a*) Describe all statistical methods, including those used to control for confounding | 12-13 | 1. Descriptive statistics were used to process the variables manifested with number (n), percentage (%), mean and standard deviation (SD).  2. For continuous variables, the normal distributions were testified by both the probability-probability plot (P-P plot) and Kolmogorov-Smirov (K-S) test.  3. Independent sample t-test and one-way analysis of variance (ANOVA) were utilized to evaluate the variations concerning the demographics and clinical variables.  4. Pearson’s correlation was applied to evaluate correlations among continuous variables.  5. Hierarchical regression analysis was applied to explore the associations of PSS, hope, optimism, general self-efficacy, resilience with fatigue along with the moderating roles of internal psychological variables on the PSS-fatigue association.  6. Asymptotic and resampling strategies were employed to explore whether hope, optimism, general self-efficacy and resilience mediated the PSS-fatigue association. A bias-corrected and accelerated 95% confidence interval (BCa 95% CI) for each mediation was conducted with the boot­strap estimate on a basis of 5,000 bootstrap samples, in which exclusion of 0 implied the variables indeed contributed as mediating factors to take effect.  All the confounders were treated as controlled variables statistically to control for confounding. |
|  |  | (*b*) Describe any methods used to examine subgroups and interactions | - | None |
|  |  | (*c*) Explain how missing data were addressed | - | There are no missing data among the RA patients enrolled. |
|  |  | (*d*) *Cohort study*—If applicable, explain how loss to follow-up was addressed  *Case-control study*—If applicable, explain how matching of cases and controls was addressed  *Cross-sectional study*—If applicable, describe analytical methods taking account of sampling strategy | - | None |
|  |  | (*e*) Describe any sensitivity analyses | - | None |
| Results | | | | |
| Participants | 13* | (a) Report numbers of individuals at each stage of study—eg numbers potentially eligible, examined for eligibility, confirmed eligible, included in the study, completing follow-up, and analysed | 8 | Potentially eligible:325 patients; examined for eligibility: 325 patients; confirmed eligible: 325 patients;  finally included in the study and analysed: 305 patients. |
|  |  | (b) Give reasons for non-participation at each stage | 8 | 1. At the initial stage, there were 12 respondents refused to cooperate.  2. And there were 8 respondents which missing information accounting for more than 30% removed subsequently. |
|  |  | (c) Consider use of a flow diagram | - | There are no flow diagrams. |
| Descriptive data | 14* | (a) Give characteristics of study participants (eg demographic, clinical, social) and information on exposures and potential confounders | 13-16 | Patients with age ranging from 41-60 accounted for the most of RA patients. Females accounted for more than 3 times than males. The married/cohabited people accounted for 87.54%. Only 21.31% holding junior college degree or above. Patients under unemployment accounted for the most with 61.97%.  Detailed information was displayed in Table1. |
|  |  | (b) Indicate number of participants with missing data for each variable of interest | 8 | There were 8 respondents which missing information accounting for more than 30%. |
|  |  | (c) *Cohort study*—Summarise follow-up time (eg, average and total amount) | - | Our research is a cross-sectional study. |
| Outcome data | 15* | *Cohort study*—Report numbers of outcome events or summary measures over time | - | *-* |
|  |  | *Case-control study—*Report numbers in each exposure category, or summary measures of exposure | *-* | *-* |
|  |  | *Cross-sectional study—*Report numbers of outcome events or summary measures | 14-22 | Four(Table1-Table4) |
| Main results | 16 | (*a*) Give unadjusted estimates and, if applicable, confounder-adjusted estimates and their precision (eg, 95% confidence interval). Make clear which confounders were adjusted for and why they were included | 13-22 | All the confounders were treated as controlled variables statistically to control for confounding. Detailed information was displayed among Table1-4. |
|  |  | (*b*) Report category boundaries when continuous variables were categorized | 11-12 | Age was classified as 3 groups: 40, 41-60 and >60 years old.  Duration of early morning stiffness (EMS) was categorized as: <0.5, 0.5-1 and >1 hour.  DAS28CRP was sorted into 4 groups: clinical remission ≤2.6, low level of disease activity 2.6-3.2, moderate level 3.2-5.1 and high level >5.1 according to the criteria.  Other continuous variables were treated just as continuous variables without any groupings. |
|  |  | (*c*) If relevant, consider translating estimates of relative risk into absolute risk for a meaningful time period | - | None |

Continued on next page

| Other analyses | 17 | Report other analyses done—eg analyses of subgroups and interactions, and sensitivity analyses | - | None |
| --- | --- | --- | --- | --- |
| Discussion | | | | |
| Key results | 18 | Summarise key results with reference to study objectives | 23-25 | Fatigue is indeed serious in RA.  1. PSS had a negative association with fatigue significantly, accounting for almost half of the variance concerning its predicting role.  2. Hope, optimism and resilience, contributed as mediators, affected the PSS-fatigue association accounting for 6.7% of the variance in predicting fatigue.  3. Moreover, resilience also moderated on the PSS-fatigue association positively. 4. Self-efficacy was negatively correlated to fatigue in univariate analysis and didn’t have statistical significance in the regression analysis. |
| Limitations | 19 | Discuss limitations of the study, taking into account sources of potential bias or imprecision. Discuss both direction and magnitude of any potential bias | 26 | 1. Self-report measures were applied to evaluate patients’ conditions and some of them fulfilled the questionnaires with the help of others due to illiteracy, which might lead to recall and reporting bias affecting the associations among variables.  2. Our research was conducted in the northeast of China with multicenters, but whether the results adapted to other population with different cultural backgrounds required to be further studied.  3. The cross-sectional method applied in our research just provided general conditions of patients at the exact time point when investigated restricting the evaluation of casual links among the variables. |
| Interpretation | 20 | Give a cautious overall interpretation of results considering objectives, limitations, multiplicity of analyses, results from similar studies, and other relevant evidence | 23-26 | Fatigue is a severe symptom among RA patients. PSS, hope, optimism and resilience could contribute as effective recourses to alleviate fatigue, which PSS has the greatest effect. Only resilience positively moderates the PSS-fatigue association. Hope, optimism and resilience could act as partial mediators in the association between PSS and fatigue. Adequate social support and positive psychological interventions targeted hope, optimism and resilience should be attached great importance to alleviate fatigue symptom among RA patients. Detailed information was displayed in the part of Discussion. |
| Generalisability | 21 | Discuss the generalisability (external validity) of the study results | 26 | Our research was conducted in the northeast of China with multicenters, but whether the results adapted to other population with different cultural backgrounds required to be further studied. |
| Other information | |  | | |
| Funding | 22 | Give the source of funding and the role of the funders for the present study and, if applicable, for the original study on which the present article is based | - | The funding was provided by the program of the Science & Technology Department in Liaoning Province. |

*Give information separately for cases and controls in case-control studies and, if applicable, for exposed and unexposed groups in cohort and cross-sectional studies.

**Note:** An Explanation and Elaboration article discusses each checklist item and gives methodological background and published examples of transparent reporting. The STROBE checklist is best used in conjunction with this article (freely available on the Web sites of PLoS Medicine at http://www.plosmedicine.org/, Annals of Internal Medicine at http://www.annals.org/, and Epidemiology at http://www.epidem.com/). Information on the STROBE Initiative is available at www.strobe-statement.org.
